# Phylogenetic Analyses and Characterization of RNase X25 from *Drosophila melanogaster* Suggest a Conserved Housekeeping Role and Additional Functions for RNase T2 Enzymes in Protostomes

**DOI:** 10.1371/journal.pone.0105444

**Published:** 2014-08-18

**Authors:** Linda Ambrosio, Stephanie Morriss, Ayesha Riaz, Ryan Bailey, Jian Ding, Gustavo C. MacIntosh

**Affiliations:** 1 Roy J. Carver Department of Biochemistry, Biophysics and Molecular Biology, Iowa State University, Ames, Iowa, United States of America; 2 Interdepartmental Genetics Graduate Program, Iowa State University, Ames, Iowa, United States of America; 3 Children's Hospital Boston, Harvard Medical School, Boston, Massachusetts, United States of America; Rosalind Franklin University, United States of America

## Abstract

Ribonucleases belonging to the RNase T2 family are enzymes associated with the secretory pathway that are almost absolutely conserved in all eukaryotes. Studies in plants and vertebrates suggest they have an important housekeeping function in rRNA recycling. However, little is known about this family of enzymes in protostomes. We characterized RNase X25, the only RNase T2 enzyme in *Drosophila melanogaster*. We found that RNase X25 is the major contributor of ribonuclease activity in flies as detected by *in gel* assays, and has an acidic pH preference. Gene expression analyses showed that the *RNase X25* transcript is present in all adult tissues and developmental stages. *RNase X25* expression is elevated in response to nutritional stresses; consistent with the hypothesis that this enzyme has a housekeeping role in recycling RNA. A correlation between induction of *RNase X25* expression and autophagy was observed. Moreover, induction of gene expression was triggered by oxidative stress suggesting that RNase X25 may have additional roles in stress responses. Phylogenetic analyses of this family in protostomes showed that RNase T2 genes have undergone duplication events followed by divergence in several phyla, including the loss of catalytic residues, and suggest that RNase T2 proteins have acquired novel functions. Among those, it is likely that a role in host immunosuppression evolved independently in several groups, including parasitic Platyhelminthes and parasitoid wasps. The presence of only one RNase T2 gene in the *D. melanogaster* genome, without any other evident secretory RNase activity detected, makes this organism an ideal system to study the cellular functions of RNase T2 proteins associated with RNA recycling and maintenance of cellular homeostasis. On the other hand, the discovery of gene duplications in several protostome genomes also presents interesting new avenues to study additional biological functions of this ancient family of proteins.

## Introduction

Members of the RNase T2 family of enzymes catalyze endonucleolytic RNA cleavage via a 2′-3′-cyclic phosphate intermediate [1]. These ribonucleases (RNases) are found ubiquitously, with RNase T2 genes in genomes of most eukaryotes, many bacteria, as well as some viruses [1], [2]. Although primary sequence identity between eukaryotic and prokaryotic enzymes is low, there are conserved secondary structures that contain key core hydrophobic residues associated with the RNase T2 active site [2], [3]. All characterized RNase T2 family members consist of a central four-stranded antiparallel β-sheet (strands β1, β2, β4 and β5), a small two-stranded antiparallel β-sheet (β3 and β7), and three α-helices (αB, αC, αD), with the catalytic site of the enzyme residing mainly within strands β2 and β5 and helix αC (Kurihara et al. 1996; Tanaka et al. 2000; Rodriguez et al. 2008). Two histidine residues within β2 and αC, together with surrounding residues form the conserved active site (CAS) motifs CAS I and CAS II [2]. Importantly, each histidine is a direct participant in the acid-base catalysis mechanism that enables the transphosphorylation and hydrolysis reactions of RNase T2 enzymes [1], [4].

These ancient ribonucleases are secreted or targeted to membrane-bound intracellular compartments (lysosomes and vacuoles) where they degrade single stranded RNAs. Long known for their function in gametophytic self-incompatibility, and as part of the response to phosphate starvation in plants [2], [5], the RNase T2 family has been recently shown to play distinctly different developmental and physiological roles in plants and animals. Recent insights from *Arabidopsis thaliana* and zebrafish indicate that conservation of the RNase T2 family in all eukaryotes may be related to an important housekeeping function carried out by these enzymes, which includes recycling of ribosomal RNAs [6], [7]. A ribophagy-like pathway is thought to mediate this turnover of rRNAs in normal, non-stressed cells [8], which is essential to maintain cellular homeostasis. Additionally, in *Saccharomyces cerevisiae* and *Tetrahymena thermophila*, the enzymatic activities of RNase T2 proteins have been associated with cleavage of mature tRNAs to produce tRNA halves in response to starvation and oxidative stress [9], [10]. The significance of the accumulation of these degradation intermediates is unknown, although it has been suggested that they may play a signaling role in the maintenance of cellular homeostasis [5]. Alternatively, they may accumulate as a consequence of targeted degradation of the translation machinery during stress conditions that leads to suppression of cell division [10]. Interestingly, a different ribonuclease carries out tRNA cleavage function in response to stress in vertebrate cells. In this case angiogenin, a member of the vertebrate-specific RNase A family, is responsible for the accumulation of tRNA fragments [11].

At least one member of the RNase T2 family has been found in every eukaryotic genome that has been sequenced, with Trypanosomatids as the only exception [2]. High frequency of gene duplication and extensive divergence of the T2 RNases has occurred in plants [12], [13]. On the other hand, only one, well-conserved gene, is found in most vertebrate genomes [14]; and it has been proposed that RNase A members have replaced RNase T2 in several biological roles in these organisms [2], [14]. Thus, characterization of the biological role played by RNase T2 enzymes in multicellular organisms is complicated in plants and vertebrates due to the presence of potentially redundant enzymatic activities. In contrast, the *Drosophila melanogaster* genome contains only one RNase T2 gene, *RNase X25* (also known as *DmRNase-66B*), and no RNase A homolog; thus, this organism could be used as a simpler system to demonstrate the conserved function(s) of this enzyme family in animals. *RNase X25* (CG8194), located at 66A21 on chromosome 3, is 1658 nucleotides in length and encodes a single form of mRNA transcript with a 325 amino acid open reading frame [15]. A signal peptide cleavage site is anticipated between residues 21 and 22 suggesting transport of the predicted polypeptide chain to the secretory pathway. In addition, two asparagine residues (positions 214 and 231) and a threonine (residue 34) may serve as N- and O-glycosylation sites, respectively. N-glycosylation is the most common modification found for the RNase T2 family, while a few cases of O-glycosylation have been observed for fungal enzymes (reviewed in [2]).

As a first step towards understanding the role of RNases T2 in animals, biochemical analyses and gene expression studies were initiated in the fruit fly *D. melanogaster*. RNase T2 activity was detected in all *Drosophila* life cycle stages examined, and this correlated well with *RNase X25* gene expression patterns. Furthermore, *RNase X25* gene expression levels were responsive to nutritional and oxidative stress as determined by the accumulation of *RNase X25* mRNAs in larvae starved for nutrients or exposed to wheat germ agglutinin (WGA), or hydrogen peroxide. A correlation between induction of autophagy and increased RNase X25 expression and activity was also observed in response to starvation. Finally, we used phylogenetic analyses to shed light on the evolution of the RNase T2 family of ribonucleases in protostomes and found evidence for gene duplications followed by divergence and the potential acquisition of new functions in several phyla, in contrast to the pattern observed in most deuterostomes. Together, these analyses suggest that RNase X25 carries out a conserved housekeeping function as proposed for other RNases T2 in plants and animals, and that *Drosophila*, with a single RNase T2 gene, is a good eukaryotic model system in which to investigate the role of RNases T2 in the process of ribophagy. The discovery of gene duplications in several protostome genomes also presents interesting new avenues to study additional roles of this ancient family of proteins.

## Material and Methods

### *D. melanogaster* strains, and tissue preparations

In this study the *Drosophila melanogaster* strain *w^1118^*/*w^1118^* with two wild type *RNase X25* genes and *w^1118^*/*w^1118^*; *Df(3L)Excel6279/+* (denoted *Df(3L)Excel6279/+* in the text), with one wild type *RNase X25* gene were raised at 25°C on standard cornmeal media.

For staged embryo collections, females were placed in collecting bottles and eggs were gathered after aging from molasses-agar plates dusted with yeast.

### Stress treatments

To provide standardized non-crowded growing conditions prior to stress treatments, 43 *w^1118^/w^1118^* embryos (0–2 hrs) were gently transferred onto Formula 4–24 instant blue *D. melanogaster* diet (363.6 mg/1.625 µl H_2_O; Carolina Biological Supply, Burlington, NC, USA), that had been placed into a small petri dish (60×15 mm). Baker's yeast was not sprinkled on this medium. Petri plates were placed in an incubator at 22°C and 80% humidity for 128 hours. Then 25 young, newly molted third instar larvae were gently transferred from each plate to either control *D. melanogaster* diet, or experimental media consisting of *D. melanogaster* diet containing 1% unconjugated wheat germ agglutinin (Vector Laboratories, Burlingame, CA, USA), or hydrogen peroxide at 0.1% [w/w] or 0.5% [w/w]. For starvation conditions larvae were placed onto PBS-saturated Whatman 1 filter paper. After 14 hours, larvae were collected, frozen at −80°C and stored for further processing. For detection of autophagy in fat body cells of starved and fed control larvae, embryos were placed onto Bloomington's Drosophila Stock Center cornmeal/molasses/yeast soft media, sprinkled with Baker's yeast, and subsequently processed as described above. LysoTracker Red DND-99 (Life Technologies, Carlsbad, CA, USA) staining of lysosomes and autolysosomes, and Hoeschst 33342 (Thermo Fisher Scientific Inc, Rockford, IL, USA) staining of DNA was performed as described by Scott et al. [16] and Juhasz and Neufeld [17]. Stained fat body lobes were imaged in PBS using a Zeiss Axio Imager.Z2 microscope equipped with AxioCam HR digital camera using a LD Plan-Neofluar 40x/0.6 objective lens and ZEN imaging software.

### Protein extracts and RNase activity assays

Protein was prepared from flies at different stages or collected from stress experiments, using approximately 100 mg of each sample. The material was homogenized in 1.5 ml eppendorf tubes and protein extractions were performed as described by Hillwig et al [14], using the protease inhibitor cocktail Complete Mini EDTA Free (Roche Diagnostics, Indianapolis, IN, USA) or Protease Inhibitor Cocktail P8340 (Sigma-Aldrich, St. Louis, MO, USA). *In gel* RNase activity assays were performed following the protocol used by Yen and Green [18] using high molecular weight Torula Yeast RNA (Sigma-Aldrich) as substrate, loading 20–80 µg of protein per lane. After running and washes, gels were incubated at pH 6.0 or 7.0, as indicated in the figures. SDS-PAGE was run in parallel for each sample as loading and quality control, also using 20 µg of protein per lane, and then stained with Coomassie Brilliant Blue. Experiments were repeated at least 3 times. A representative gel is shown.

### qPCR analysis

RNA was extracted from 100 mg of sample using Trizol (Fisher) according to manufacturers' instructions. RNA was DNase-treated using Turbo DNA-free (Ambion), and cDNA was synthesized using the iScript Select cDNA Synthesis kit (Bio-Rad), also following manufacturers' instructions for each procedure. qPCR was completed on a Stratagene MX4000 using the Absolute qPCR with SYBR Green + Rox kit (Fisher Scientific) according to manufacturers' instructions. The transcript of ribosomal protein L32 gene (*RPL-32)* was used as the control for data normalization, using the Pfaffl method [19]. Primers used for *RNase X25* were: Forward (5′-3′): TCCACGCCCCTCAGCGACATA, and Reverse (5′-3′): ACGCCAAGTGAGCCCCTGCT; for *RPL-32*: Forward (5′-3′): TGGGACACCTTCTTCAAGAT, and Reverse (5′-3′): CAGGCGACCGTTGGGGTTG; for *Atg5*: Forward (5′-3′): ATCTGGGAGGGCCAGATAGG, and Reverse (5′-3′): TAGCTCCTTGGAGTTGAGCTTG; for *Amyrel*: Forward (5′-3′): GATCTAGAGTACATCTACAGCAGCC, and Reverse (5′-3′): ACTTGTAGTTCAGCACGGCA; and for *Lip3*: Forward (5′-3′): GCCTATTTCTGATTGCGGTGAG, and Reverse (5′-3′): AGTACTTGTGCGCCTTGGAG.

Experiments were performed using triplicates, and repeated 3 times using independent samples. Statistical significance of the differences between treatments was determined using *t*-test. Graphs show averages of each sample normalized using the average value of the control sample. One star  = P<0.05, two stars  =  P<0.01.

### Phylogenetic analysis

Identification of protostome RNase T2 genes or proteins was done by BLAST searches [20] using Ensembl Genomes [21], VectorBase [22], the Genome Portal of the Department of Energy Joint Genome Institute [23], the *Clonorchis sinensis* Genome Database [24], SmedGD [25], the Hymenoptera Genome Database [26], SilkDB [27], Beetle Base [28], GeneDB [29], AphidBase [30], and the National Center for Biotechnology Information Map Viewer (http://www.ncbi.nlm.nih.gov/projects/mapview/).

Protein sequences were aligned using ClustalW2 [31] followed by manual adjustments. PAUP 4.0 software [32] was used for neighbor-joining (1,000 bootstrap replications) and parsimony analyses, using default parameters.

## Results

### Characterization of RNase activity in Drosophila development

**Figure 1:**
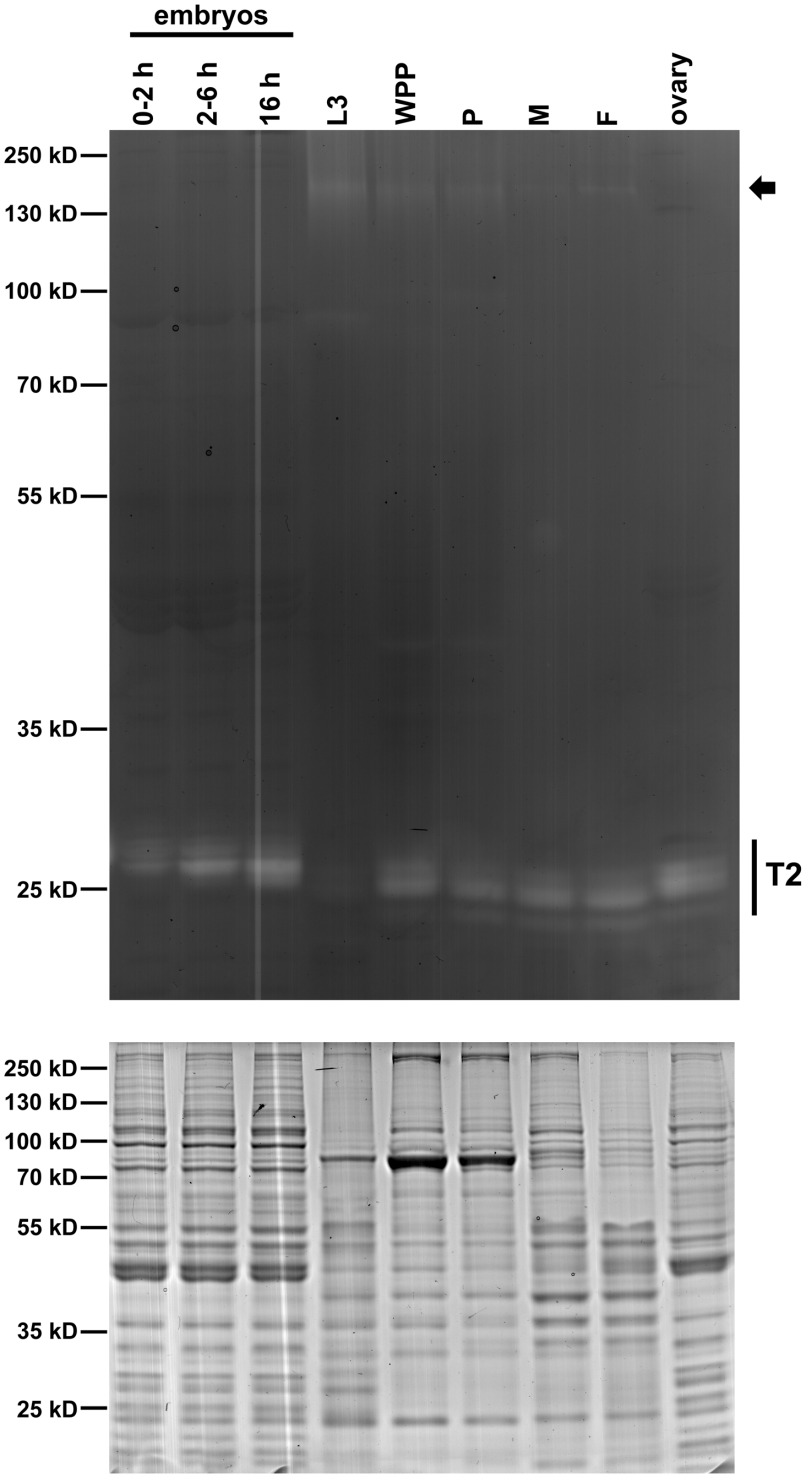
Developmental profile of *Drosophila* RNase activities. Protein extracts were produced from embryos at 0–2 hours (h), 2–6 h, and 0–16 h after egg deposition and from animals at 3rd instar larval (L3), white prepupal (WPP), pupal (P), and adult male (M) or female (F) stages of development. Ovarian tissue (ovary) was prepared from 3–5 day old females. (Upper panel) Protein was fractionated by electrophoresis through a 12% polyacrylamide gel containing 3 mg/ml Torula yeast RNA, washed to remove SDS, incubated in 100 mM Tris-HCl at pH 6.0 and stained with toluidine blue to visualize regions of nuclease activity. Low molecular weight (∼25–30 kD) activities in the size range of the RNase T2 family were detected at all developmental stages assayed. High molecular weight (∼200 kD) activities were also apparent (arrow), but absent from embryos. (Lower panel) Protein extracts were analyzed by SDS/PAGE and stained with Coomassie Blue R-250 to control for equal loading and protein integrity. Each lane in both gels contains 20 µg of protein.

The activity of the RNases with characteristics similar to the RNase T2 family, i.e. endonucleases with no sequence/base specificity, has not been characterized in *Drosophila*. To detect RNase activities in extracts from different *Drosophila* developmental stages we used a standard *in gel* activity assay that allows size separation of different proteins with RNase activity. Embryos at 0–2, 2–6, and 0–16 hr after egg deposition, as well as wandering third instar larvae, white prepupae, pupae, adult males, females, and isolated ovaries were collected. Protein extracts were prepared and analyzed for RNase activities (Figure 1, top panel). At all developmental stages, RNase activities in the apparent 25–30 kDa range, which correspond to the predicted size range of RNase T2 enzymes, were observed. It is important to note that the PAGE method used for this assay is semi-denaturing, since it includes SDS but not reducing agents, and the apparent molecular weight observed for each protein band does not necessarily correspond to the predicted mass. Detection of RNase activities with distinct molecular weights in this range may be indicative of posttranslational processing, including N- and O-glycosylation, a common posttranslational modification of RNase T2 proteins. While most stages showed a similar level of RNase activity, samples collected from early 0–2 hr embryos and third instar larvae showed lower RNase activity. This result was not due to general protein degradation since protein integrity seems evident in a Coomassie stained SDS-PAGE (Figure 1, bottom panel). In addition, a band of RNase activity at a very large apparent molecular weight (∼200 kDa) was observed primarily in third instar larvae, white prepupae, and pupae (Figure 1, top panel, arrow).

**Figure 2:**
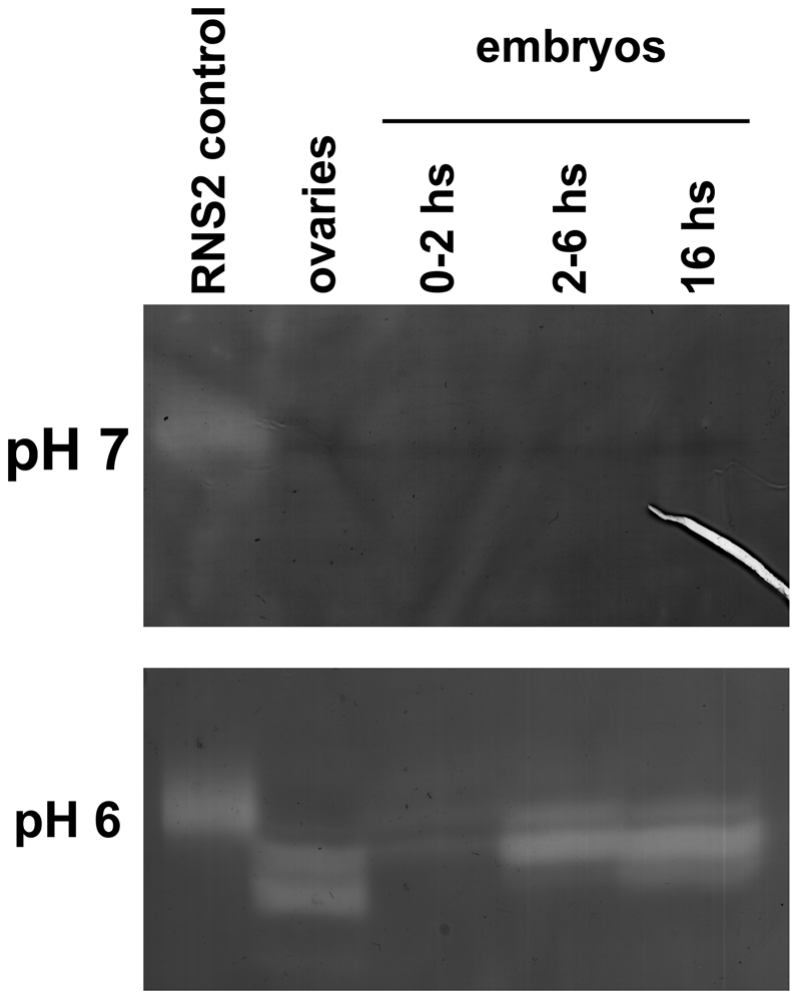
Effect of pH on *Drosophila* RNase activities. Protein extracts from ovaries and embryos were analyzed using RNase *in gel* activity assays as described in Figure 1, with incubations at neutral (pH 7.0; upper panel) and acidic (pH 6.0; lower panel) conditions. RNase activity in the size range corresponding to RNase T2 enzymes was abundant after incubation at pH 6, while almost no activity was observed at neutral pH. Each lane in both gels contains 20 µg of protein. A plant protein that is active at the two pH conditions, *Arabidopsis thaliana* RNS2 [7], was used as control.

We undertook a combined biochemical-genetics strategy to more definitely assign the RNase activity observed on our activity gels to the *RNase X25* gene product. One important defining characteristic of the T2 family of RNases in animals is their pH sensitivity and acidic preference [1], [2], [3]. Thus, we compared RNase activities in *Drosophila* ovarian and embryonic extracts using *in gel* activity assays at different pH conditions (Figure 2). At pH 7, little to no RNase activity was observed at the 25-30 kDa range, while robust activity was evident in samples from ovary and embryos at acidic pH. A large number of endo and exoribonuclease activities are predicted based on sequence analysis of the *Drosophila* genome. Classical genetic mutations in *RNase X25* are currently unavailable and RNA interference stocks without off-target effects have not been produced. Therefore, we employed a chromosomal deletion approach to determine whether a decrease in gene dose could affect the relative amount of RNase activity detected in our assays.

**Figure 3:**
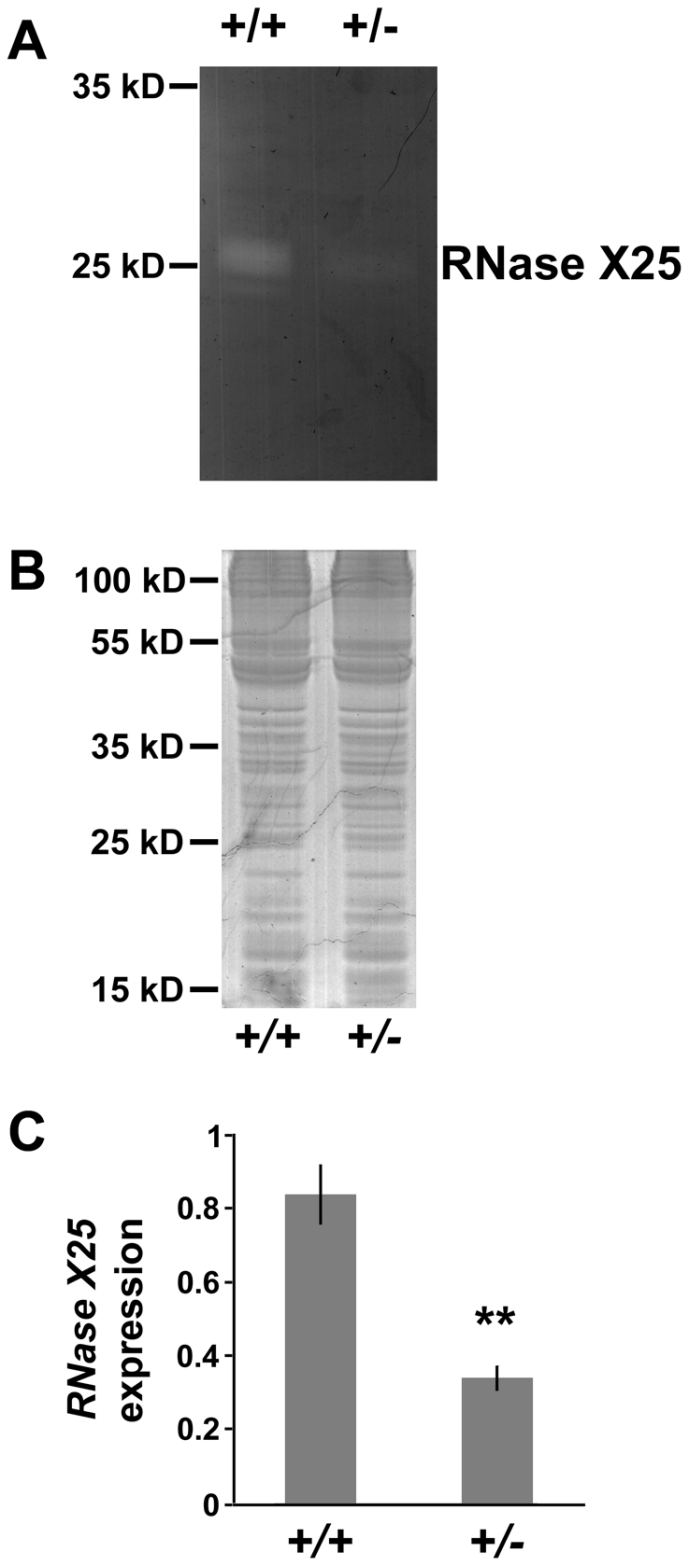
Reduced RNase activity and expression correlates with reduced *RNase X25* gene dose. Ovarian extracts were prepared from wild type control (*+/+*), or deletion mutant *Df(3L)Excel6279/+* females (*+/−*), carrying two or one copy of the *RNase X25* gene, respectively. Protein samples were analyzed using (A) *in gel* RNase activity assay, or (B) standard SDS/PAGE analysis. Compared to the control (*+/+*), RNase activity was reduced in ovaries dissected from females with one copy of the *RNase X25* gene (*+/−*). Each lane in both gels contains 20 µg of protein. (C) RNA was isolated from ovaries and qPCR quantification of the relative level of *RNase X25* mRNAs in these samples was carried out using the ribosomal protein L3 (*RpL3*) transcript as internal standard control for normalization. *RNase X25* expression levels were reduced in tissue samples from mutant *Df(3L)Excel6279/+* females (*+/−*), compared to control females (*+/+*). Data are representative of 3 independent experiments and are means and S.E. of triplicates. **, *P*<0.01 (*t*-test).

The *Df(3L)Excel6279* chromosome was chosen, with deficiency break points mapped to 66A17 and 66B5. This is the smallest known deletion that removes the *RNase X25* gene located at position 66A21. Importantly, *RNase X25* is the only RNase encoding gene that lies between the breakpoints of the *Df(3L)Excel6279* chromosome. RNA and protein extracts were produced from ovarian tissue from either a wild type (*+/+*) genetic background with two *RNase X25* gene copies or the *Df(3L)Excel6279/+* background with one *RNase X25* gene copy. A homozygous mutant line could not be obtained, since homozygous deletions of this region are lethal. Quantitative RT-PCR analysis indicated approximately one-half of wild-type *RNase X25* mRNA levels were detected for the *Df(3L)Excel6279/+* ovaries (Figure 3C). Furthermore, a corresponding decrease in RNase activity was observed for the 25–30 kDa bands in *Df(3L)Excel6279/+* extracts (Figure 3A), when similar amounts of protein were examined for wild-type and heterozygous deletion mutants (Figure 3B). These results strongly suggested that the enzymatic activity observed by our *in gel* analysis was, in fact, RNase T2 activity, encoded by the *Drosophila RNase X25* gene.

**Figure 4:**
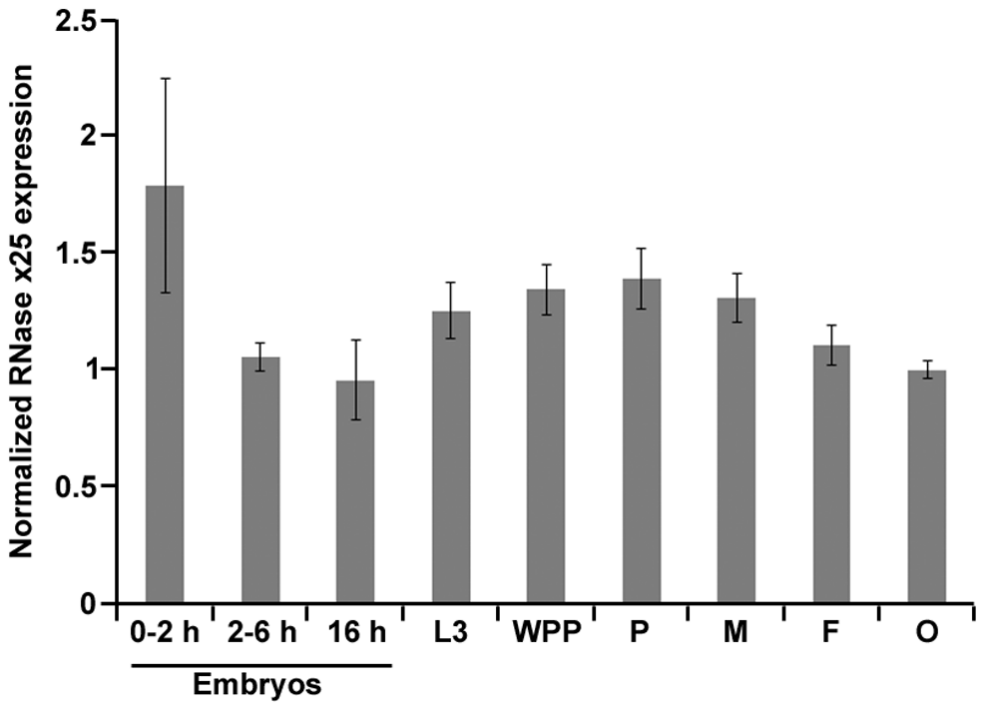
Developmental profile of *RNase X25* transcript accumulation. RNA was isolated from embryos at 0–2 h, 2–6 h, and 0–16 h after egg deposition and from animals at 3rd instar larval (L3), white prepupal (WPP), pupal (P), and adult male (M) or female (F) stages of development. Ovarian tissue (O) was prepared from 3–5 day old females. qPCR quantification of the relative level of *RNase X25* mRNAs in these samples was carried out using the ribosomal protein L3 (*RpL3*) transcript as internal standard control for normalization. *RNase X25* expression was detected at all stages analyzed. Data are representative of 3 independent experiments and are means and S.E. of triplicates.

Total RNA was also isolated from developmental samples, and *RNase X25* expression was analyzed using quantitative real time PCR (qRT-PCR) studies (Figure 4). This analysis indicated that *RNase X25* transcripts are present in all the stages analyzed, showing constitutive expression throughout *Drosophila* development. No significant stage-specific differences in mRNA accumulation were apparent in this experiment. Our gene expression analyses correspond well with expression data obtained from genome-wide transcriptome analyses deposited in FlyBase (http://flybase.org). Moreover, data obtained from the modENCODE [33] and FlyAtlas [34] databases indicated that *RNase X25* expression is constitutive for all tissues of the fly at the 3^rd^ instar larva and adult developmental stages with tissue specific expression ranging from very low to high levels (Figures S1 and S2). It is intriguing that early embryos and third instar larvae, the two samples with low RNase activity, had at least as much expression of *RNase X25* as samples with high activity. The discrepancy between enzymatic activity and mRNA accumulation could suggest that RNase X25 is postrancriptionally or posttranslationally regulated.

### *RNase X25* expression is altered by stress

In addition to a general housekeeping function in rRNA recycling, the RNase T2 family of enzymes is thought to play specialized roles in unicellular and multicellular eukaryotes. In yeast and *Tetrahymena*, RNase T2 activity is responsible for the cleavage of mature tRNAs to produce tRNA halves [9], [10] during the response to oxidative stress or amino acid starvation. Several microarray and RNAseq reports on the fly's transcriptional response to a variety of stresses are available in the literature and public databases. However, results related to the effect of starvation on *RNase X25* are not clear (Table S1). To begin exploring the possibility that RNase T2 may play a role in the fruit fly's response to nutritional and oxidative stress, we determined whether *RNase X25* gene expression levels were altered after exposure to these pressures. It was also reported by Li *et al*. [35] that accumulation of *RNase X25* mRNAs was altered in *Drosophila* larval midgut tissue after animals were fed a diet supplemented with wheat germ agglutinin (WGA). Thus, we also determined if a change in *RNase X25* expression levels could be detected in whole animal extracts after larval ingestion of WGA (1% w/w).

**Figure 5:**
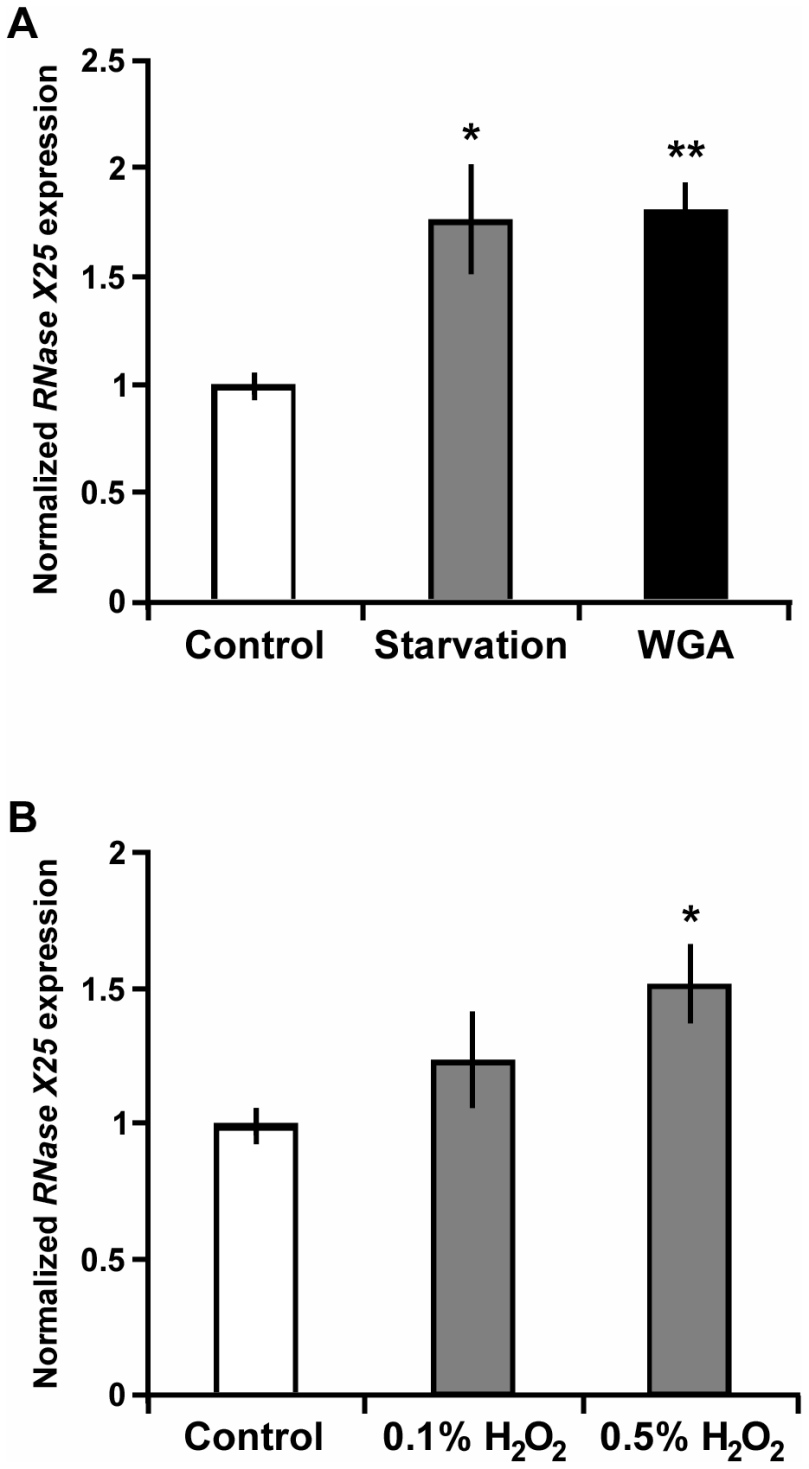
*RNase X25* gene expression is regulated by nutritional and oxidative stress. RNA was isolated from whole 3^rd^ instar larvae, 14 h after transfer to control or experimental media (see Materials and methods). qPCR quantification of the relative level of *RNase X25* mRNAs in these samples was carried out using the ribosomal protein L3 (*RpL3*) transcript as internal standard control for normalization. Increased levels of *RNase X25* transcripts were apparent in samples after (A) starvation and treatments with 1% [w/w] wheat germ agglutinin (WGA), and (B) 0.1% [w/w] or 0.5% [w/w] hydrogen peroxide. Data are representative of 3 independent experiments and are means and S.E. of triplicates. *, *P*<0.05; **, *P*<0.01 (*t*-test).

Whole animal extracts were prepared for molecular analysis from third instar larvae fed a control diet or subjected to starvation for 14 h (see Materials and Methods). As shown in Figure 5A, the accumulation of *RNase X25* mRNA transcripts increased approximately 80% for animals starved for nutrients (P<0.05) when compared to fed control larvae. It has been proposed that diets containing WGA produce a starvation-like effect on flies [35]; thus, a WGA-containing diet was also used to feed *D. melanogaster* larvae. Consistent with a starvation-like effect, a WGA diet resulted in a significant increase in *RNase X25* expression (P<0.01), similar to that observed in starved flies (Figure 5A).

We also observed an increase in *RNase X25* mRNA when larvae were fed a diet containing the oxidative stressor hydrogen peroxide (Figure 5B). In this set of experiments, the normalized *RNase X25* expression levels for whole animals exposed to 0.5% hydrogen peroxide were 50% higher (P<0.05) than observed for control animals. At a lower dosage, 0.1 %, a 20% increase in *RNase X25* mRNA was observed, although this change was not statistically significant. Thus, analysis of whole larval extracts indicated that the *RNase X25* gene was responsive to various stressors including starvation, and treatments with 1% WGA or 0.5% hydrogen peroxide. Data from microarray experiments performed by other laboratories suggest that a few other stress conditions and chemical treatments can also alter the expression of *RNase X25* (Table S1).

### Starvation, *RNase X25* Expression and Autophagy

**Figure 6:**
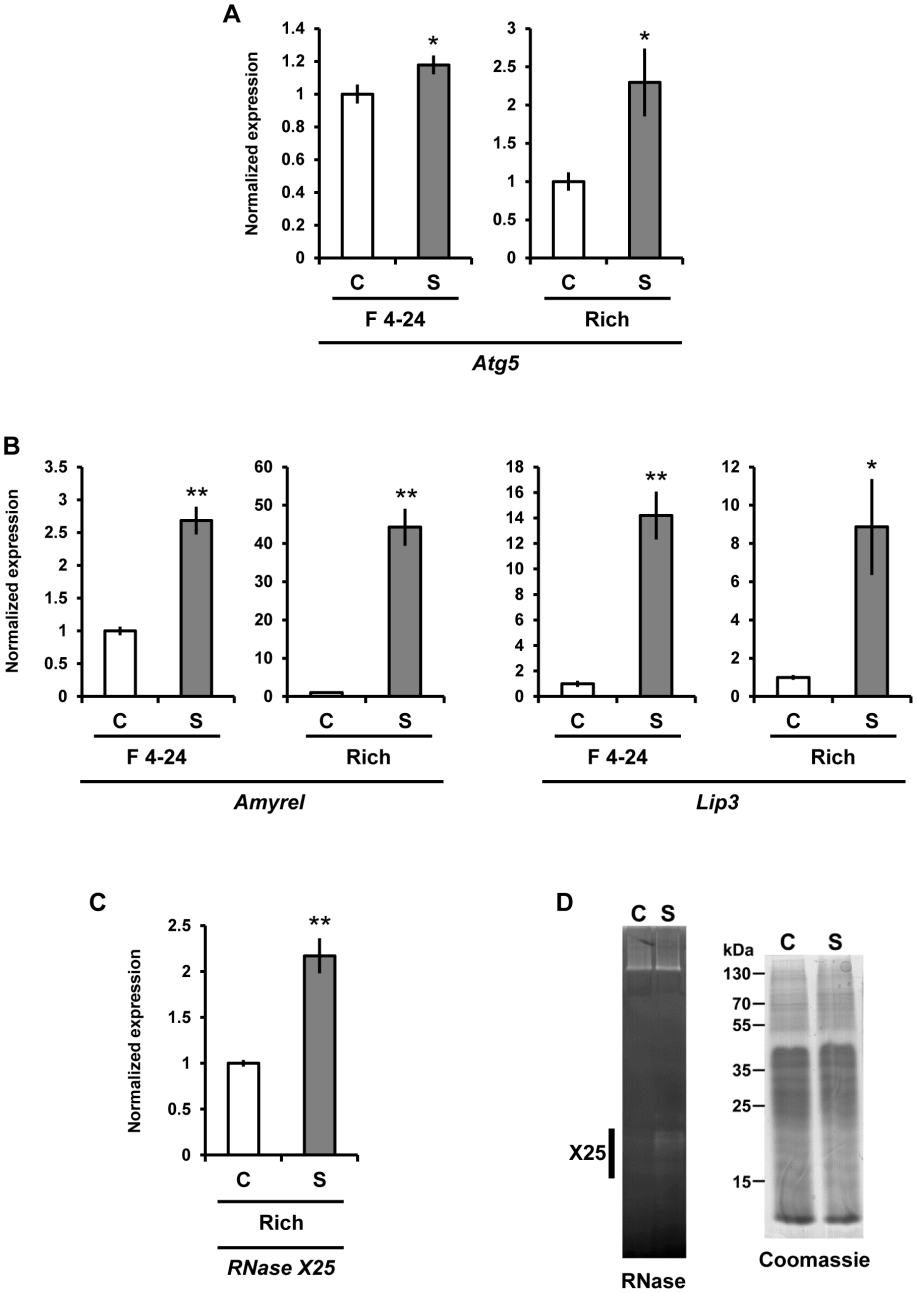
Starvation induces expression of the autophagy marker, *Atg 5* and *Amyrel, Lip3* and *RNase X25* in larvae. RNA was isolated from whole 3^rd^ instar larvae, 14 h after transfer to control (C) or starvation (S) conditions (see Materials and methods). qPCR quantification of the relative level of (A) autophagy marker *Atg5,* (B) starvation markers *Amyrel*, and *Lip3*, and (C) *RNase X25* mRNAs in these samples was carried out using the ribosomal protein L3 (*RpL3*) transcript as internal standard control for normalization. Increased levels of *Atg5, Amyrel, Lip3*, and *RNase X25* transcripts were apparent in samples after starvation as compared with fed-control animals. Data are representative of 3 independent experiments and are means and S.E. of triplicates. *, *P*<0.05; **, *P*<0.01 (*t*-test). (D) Protein extracts from 14 h starved (S) and fed-control (C) whole 3^rd^ instar larvae were analyzed using RNase *in gel* activity assays as described in Figure 1. RNase activity in the size range corresponding to RNase T2 enzymes was evident in starved as compared with fed-control animals. Each lane in both gels contains 80 µg of protein. “F 4–24” denotes extracts from animals nourished with Formula 4–24 instant *D. melanogaster* diet without yeast; “Rich” denotes extracts from animals fed a yeast rich diet (see Materials and methods).

Since starvation induces autophagy and autophagy mediated RNA degradation, we also tested whether expression of *Atg5*, which encodes a protein that participates in an ubiquitin-like protein conjugation system essential for autophagy[36], was altered in our starved larvae. We found a small but significant (P<0.05) increase in the expression of this autophagy marker in starved, as compared with fed control larval samples (Figure 6A left F 4–24 panel). Since only a low level of induced *Atg5* expression was detected in our starved samples, we used two gene markers, *Amyrel* (α-amylase related) [35] and *Lip3* (lipase) [37] to monitor the starvation response for these animals grown on Formula 4-24 instant blue *D. melanogaster* diet. As shown in Figure 6B (F 4–24 panels) significant (P<0.01) increases in the level of *Amyrel* (2.5 fold) and *Lip3* (14 fold) were apparent, indicating that the starved animals were indeed nutritionally stressed.

**Figure 7:**
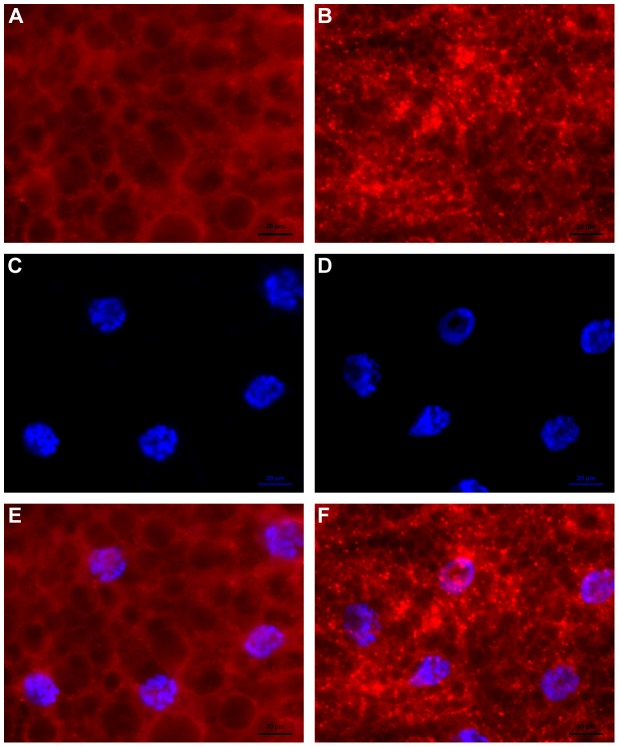
Effect of starvation on the accumulation of Lysotracker-positive vesicles in larval fat body. (A and B) A high level of bright red Lysotracker-positive vesicles accumulate in fat body cells isolated from (B) 14 h starved 3^rd^ instar larvae, with few observed for (A) fed-control larvae. (C and D) Hoescht 33342 staining of DNA, and (E and F) merged images. Scale bar = 20 µm.

Next, we followed autophagy by examining the formation of Lysotracker-positive vesicles in larval fat body, as previously described by Jimenez-Sanchez et al. [38]. For animals nourished with Formula 4–24 instant *D. melanogaster* diet, very high, but diffuse accumulation of Lysotracker was found for both fed control and starved larval fat body, complicating the interpretation of results (data not shown). The diet of these animals was not supplemented with Baker's yeast, an important and major source of nutrients for Drosophila larvae [39]. However, for 3^rd^ instar larvae growing on rich media containing yeast (see Materials and Methods), after a 14-hour starvation period, characteristic Lysotracker-positive vesicles were observed in fat body cells (Figure 7B, D and F), with little to no puncta visible for fed control animals (Figure 7A, C and E). Importantly, quantitative RT-PCR analysis demonstrated that a significantly (P<0.01) higher level of *Atg5* mRNA transcripts was present after 14 hours of starvation, as compared with those from non-starved control animals in rich media-fed larval samples (Figure 6A), confirming the induction of autophagy in these nutritionally stressed animals. The starved state of these animals was verified by the presence of significantly (P<0.01) higher levels of both *Amyrel* and *Lip3* mRNA transcripts, as compared with fed-control larvae (Figure 6B). Finally, the level of *RNase X25* gene expression and enzymatic activity were probed and found to be at higher levels for starved compared with fed-control animals (Figure 6C and D). Together, these results suggest that the autophagy process is concomitantly induced with an increase in *RNase X25* mRNA expression and enzymatic activity after starvation.

### Phylogenetic analysis of T2 RNase genes in protostomes

Phylogenetic analyses of deuterostomes and plants suggested that RNase T2 enzymes carry out an essential housekeeping role that justifies their presence in all eukaryotic genomes [2]. In addition, the RNase T2 family in plants has undergone many gene duplication events followed by gene sorting and diversification, which led to the acquisition of new biological roles [13], but this diversification was not been observed in the animal genomes so far analyzed [14], which did not include almost any protostome. The presence of only one *RNase T2* gene in the *Drosophila* genome also seemed to confirm that only one gene is present in animal genomes; nevertheless, the recent availability of a large number of fully sequenced protostome genomes led us to perform a search for members of the RNase T2 family in those genomes, followed by phylogenetic analyses.

**Figure 8:**
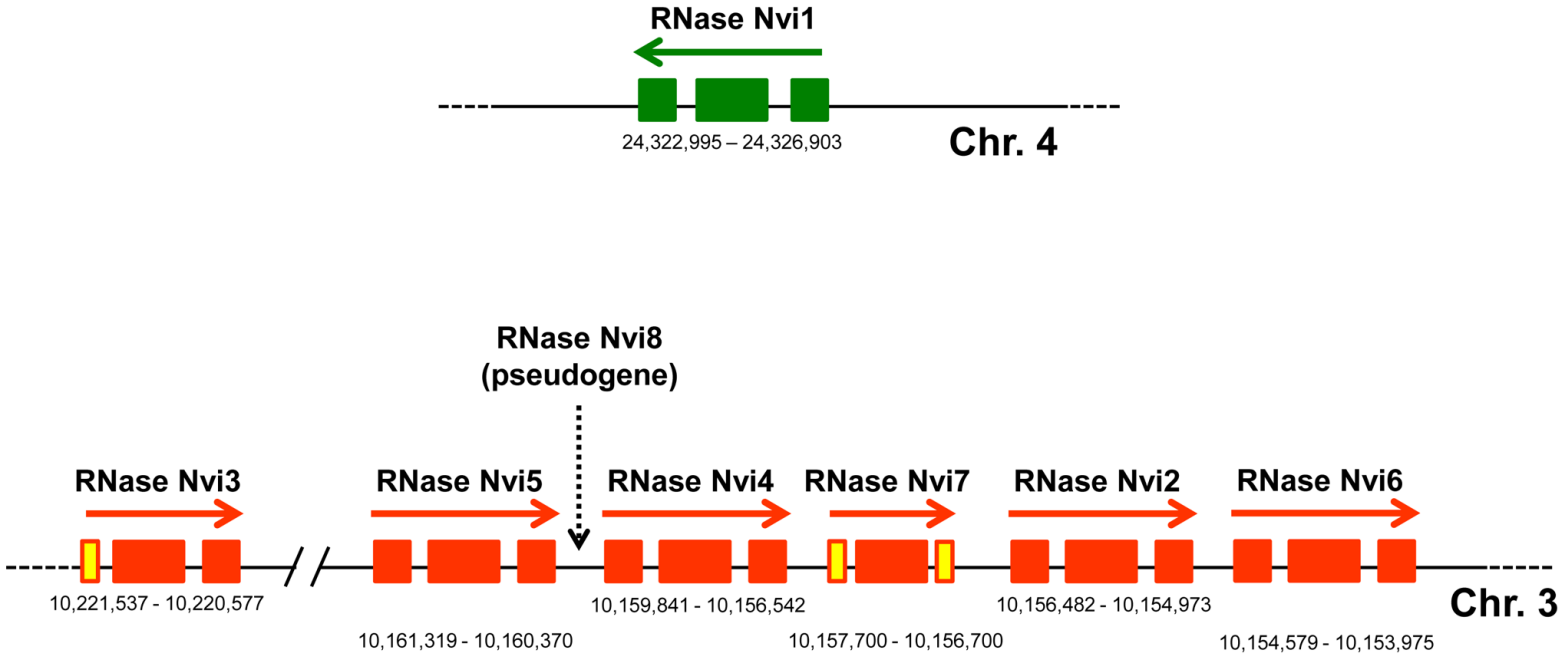
Genomic organization of the *RNase T2* genes found in the *Nasonia vitripennis* genome. Genes belonging to the RNase T2 family were identified by homology searches of the wasp genome. Boxes represent exons. The gene more closely related to other insect RNase T2 genes is shown in green. Genes with more divergence are shown in orange. Yellow boxes indicate exons with uncertain boundaries. Numbers below each gene are the coordinates of each gene based on the *Nasonia vitripennis* genome assembly Nvit_2.0. The location of the pseudogene is indicated but the gene is not depicted.

Extensive searches in all the available Drosophilidae genomes (Table S2) confirmed that this family possesses only one *RNase T2* gene. A similar result was obtained in extensive searches of other fully sequenced insect genomes, including seven species of ants, two bees, two bumblebees, red flour beetle, silkworm, and pea aphid (Table S2). Surprisingly, the analysis of parasitoid wasp genomes provided a different result. We identified eight *RNase T2* genes in the *Nasonia vitripennis* genome (Table S2 and Figure 8), seven that encoded for potential full-length proteins (RNase Nvi1 - RNase Nvi7) and one pseudogene (RNase Nvi8, sequence not shown). Analysis of expressed sequence tag (EST) databases indicated that at least two of these genes are expressed in *N. vitripennis* (not shown). Protein database searches also revealed that another parasitoid wasp species, *Glyptapanteles flavicoxis*, contained more than one *RNase T2* gene in its genome (Table S2).

Extending the search to other fully sequenced protostome genomes and EST databases produced similar results (Table S2). RNase T2 was absolutely conserved in all the genomes analyzed, supporting the hypothesis that these enzymes carry out an important housekeeping function. Additionally, different phyla or subgroups varied on whether a single or multiple genes were present in their genomes. Among Arthropoda, only one gene was found on most Hexapoda genomes except parasitic wasp, and only one full RNase T2 gene seems to be present in the only Crustacean genome (*Daphnia pulex*) available; however, Arachnids genomes have multiple expressed genes corresponding to this family. Nematoda and Annelida have only one T2 gene, based on the analysis of three and two full genome sequences, respectively. On the other hand, Mollusca and Platyhelminthes have multiple copies of RNase T2 genes in their genomes.

**Figure 9:**
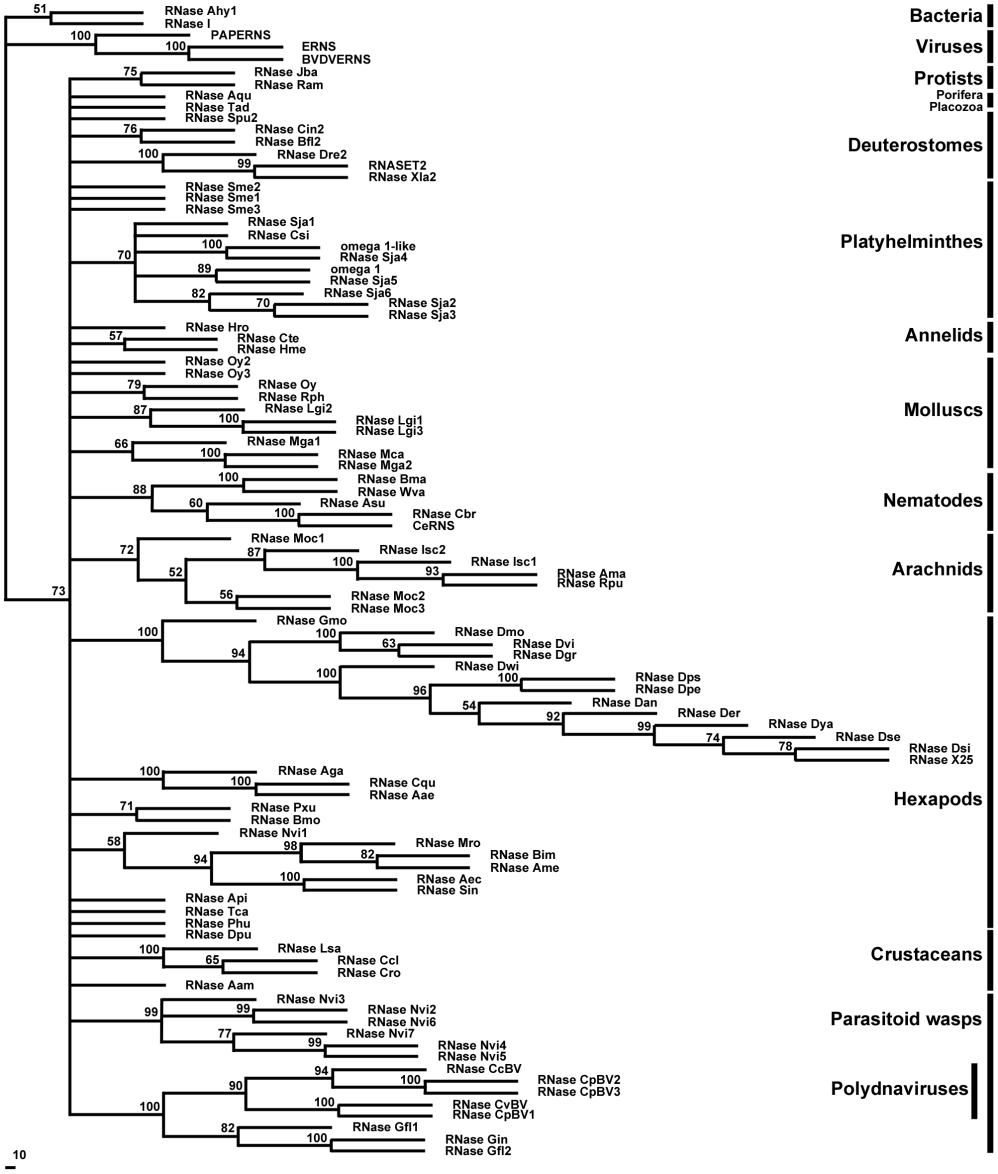
Phylogenetic tree of protostome RNase T2 proteins. Tree was obtained by the Neighbor-Joining method using only conserved regions. Bootstrap percentages (for 1,000 replications) greater than 50 are shown on interior branches. The tree was rooted using bacteria sequences. Groups discussed in the text are labeled on the right.

Neighbor-joining analysis was used to create a phylogenetic tree (Figure 9) of the protostome RNase T2 proteins extracted from full genomes and protein and EST databases. The tree showed a well-defined clade for most individual phyla, but overall it did not have good definition. This could be due to significant divergence for proteins in each phylum clade, or it could also indicate that more sequences are needed for a better resolution. A parsimony analysis showed similar results (not shown). In any case, several inferences can be made with respect to the evolution of RNase T2 proteins in protostomes. Gene duplication events seem to have happened independently in each phylum. Moreover, in some cases we found species-specific clades, in particular the one corresponding to *Nasonia vitripennis* (parasitic wasp, see below) or *Lottia gigantea* (Mollusca) that suggest that gene duplication occurred after speciation. In contrast, gene duplications in Platyhelminthes may have occurred before speciation in some cases, since it is possible to find conserved ortholog pairs for *Schistosoma japonicum* and *Schistosoma mansoni*; however proteins from *Schmidtea mediterranea* do not cluster with those from *Schistosoma* spp, suggesting either duplication after speciation or rapid divergence.

**Figure 10:**
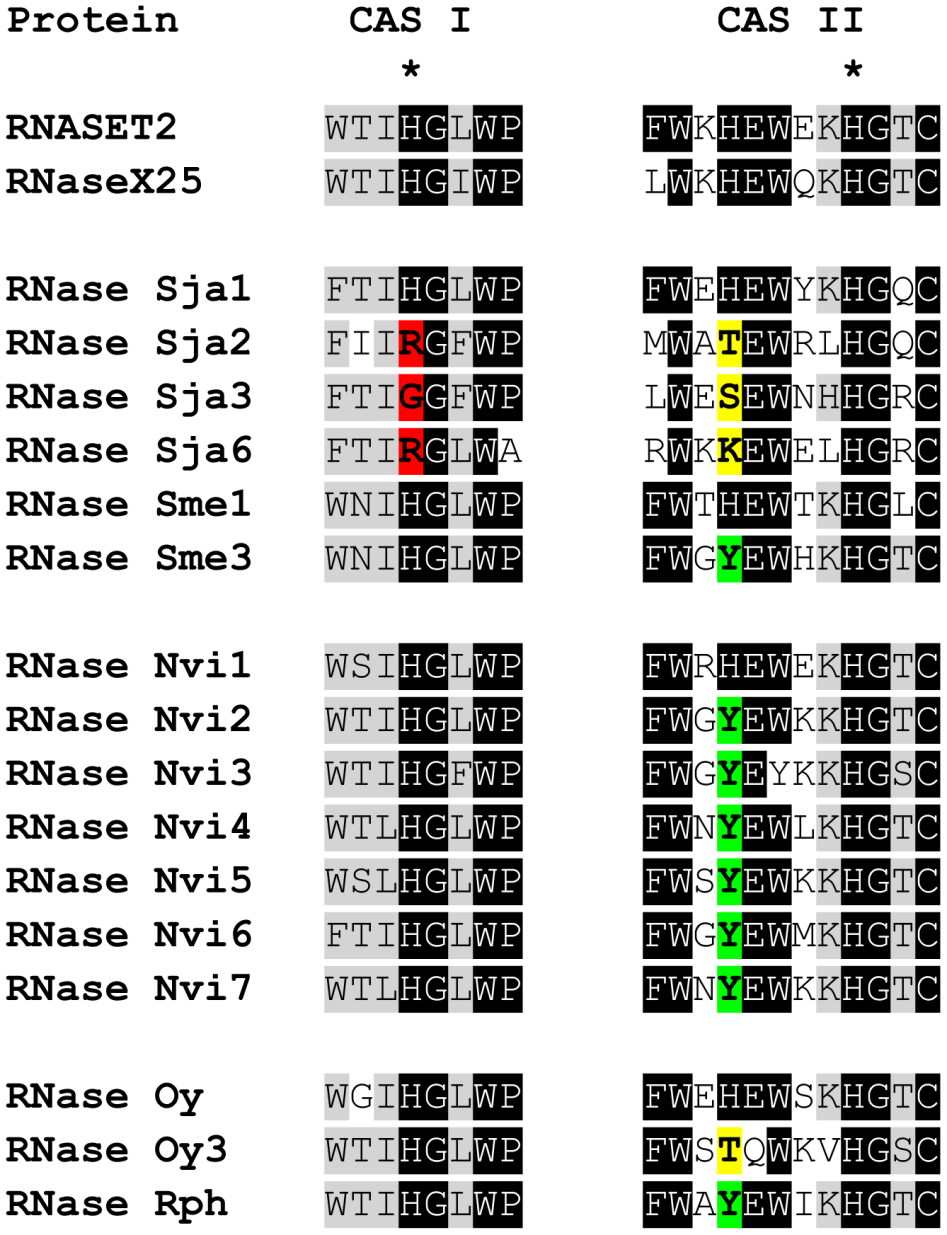
Identification of mutations in conserved active site residues in protostome RNase T2 proteins. The alignment shows the conserved CAS I and CAS II regions characteristic of RNase T2 enzymes. The catalytic histidines are marked with asterisks. Mutations in the catalytic histidine in CAS I should result in complete loss of activity (shown in red). Mutations in the additional histidine in CAS II, implicated in binding to the substrate or stabilization of the pentacovalent intermediate [2], should result in enzymes with reduced activity (shown in green or yellow). The active sites of human RNASET2 and RNase X25, two active RNases, are shown for comparison.

The parasitic wasp RNases are an interesting case. In our analysis, parasitic wasps were the only insects with multiple T2 genes in their genome. Remarkably, wasps from the Braconidae family form a symbiosis with polydnaviruses that help the insect parasitize its host [40]. Bracovirus in the Braconidae contain an RNase T2 gene in their genome, which has a role in immunosuppression of the wasp's host [41]. The RNase T2 protein predictions derived from ESTs from *Glyptapanteles flavicoxis*, a wasp from the Braconidae family, cluster with bracovirus RNases included in our phylogenetic analysis (Figure 7), indicating that these RNases are expressed from the symbiotic viral genome. However, the RNases in *N. vitripennis*, which belongs to the Pteromalidae family and does not form a symbiotic relation with polydnaviruses, form an independent clade. One *N. vitripennis* gene, *RNase Nvi1*, is included in the clade that includes the RNases from all other Hymenoptera. This gene is located in chromosome 4 in this wasp (Figure 8). The other six full-length genes (*RNase Nvi2* - *RNase Nvi7*) and a pseudogene (*RNase Nvi8*) are located in tandem in chromosome 3, and are likely the result of later gene duplications. We searched for any potential viral gene that could be linked to the RNases in chromosome 3 without success, suggesting that these RNases do not have a viral origin. The fact that these RNases form an independent cluster with strong bootstrap support suggests that these proteins diverged quickly after the initial gene duplication event. Identification of an EST from *Nasonia giraulti* (GeneBank Accession number ES622650) with 97% identity at the nucleotide level with *RNase Nvi2* indicated that this duplication event(s) should have occurred before speciation in the genus *Nasonia*. Analysis of protein sequences indicated that all the RNases from chromosome 3 have an H to Y amino acid substitution in the conserved active site II (CAS II, Figure 10) that may lead to an attenuation of enzymatic activity, as has been previously observed in other animal and plant RNase T2 proteins [2]. Substitutions in this position were also observed in some Platyhelminthes and mollusk RNases (Figure 10). Moreover, some flatworm RNases (RNase Sja2, RNase Sja3, and RNase Sja6) may have lost enzymatic activity completely, since they also have a substitution in a CAS I histidine that is essential for RNase activity [1].

## Discussion

In this work we performed an initial characterization of *Drosophila* RNase X25, the only member of the RNase T2 family present in this insect. We found constitutive expression of *RNase X25* mRNA during *Drosophila* development, and we were able to show a correlation between the main RNase activity detected in zymograms and expression of this gene in wild-type and deletion mutants, indicating that RNase X25 is a major contributor of endonuclease activity in *Drosophila* extracts. This activity has a pH optimum in the acidic range, a common characteristic of animal RNase T2 enzymes, which indicates that the active enzyme may normally be sequestered in an acidic compartment within cells to carry out its function. Animal RNase T2 proteins have been localized to lysosomes in zebrafish and humans [6], [42], and prediction of subcellular localization for RNase X25 indicated that this protein is also targeted to the secretory pathway [15].

Based on our results and data extracted from databases, RNase X25 seems to be active at all stages of development and in all larval and adult tissues investigated. This result is in agreement with those studies that have characterized the RNase T2 family in other eukaryotes [2], and suggest that, as it has been proposed for other eukaryotic RNase T2 enzymes constitutively expressed, RNase X25 may perform a housekeeping function. Absence of this constitutive RNase activity in *Arabidopsis thaliana* and zebrafish leads to accumulation of rRNA in vacuoles or lysosomes [6], [7]. Additionally, Arabidopsis plants lacking expression of *RNS2*, the housekeeping RNase T2 in this organism, show constitutive autophagy [7]. Thus, it has been proposed that the role of these RNases is to maintain normal cellular homeostasis by recycling rRNA.

A role in rRNA recycling and cellular homeostasis may also be carried out by RNase T2 enzymes in cells under nutritional stress conditions, likely through a specialized autophagy process known as ribophagy. Ribophagy, the targeted degradation of ribosomes through a mechanism that uses the autophagy machinery, has been described for yeast cells undergoing starvation [43]; and Rny1, the only RNase T2 enzyme in yeast [44], may mediate rRNA degradation under stress conditions [9]. While a direct role for RNase T2 enzymes in ribophagy has not been established, their participation in this process has been suggested for plants, animals, and unicellular eukaryotes [6], [8], [10]. Moreover, several plant *RNase T2* genes are induced under conditions of phosphate starvation, probably as a mechanism to scavenge nutrients [2], and at least two *Tetrahymena RNase T2* genes are also induced by starvation conditions [45]. We observed that expression of *RNase X25* is significantly induced in fly larvae subjected to starvation or fed WGA. Concomitant with this response we could also observe an increase in the expression of *Atg5*, which encodes one of the core components of the autophagy machinery that has been previously shown to be induced by starvation in *Drosophila* ovaries [46], and the robust appearance of Lysotracker-positive vesicles in larval fat body cells, marking lysosomes and autolysosomes participating in the autophagy process [16], [38]. Induction of *RNase X25* by nutritional stresses and evidence of autophagy may indicate that this enzyme also has a role in cellular homeostasis through recycling of cellular RNAs.

Data from a genome-wide microarray analysis of mRNA expression had previously identified *RNase X25* as one of 61 transcripts differentially expressed when animals were fed a 1% WGA diet [35]. In that study, a 9-fold increase in *RNase X25* transcript levels was observed for midgut tissue dissected from third instar larvae. Since whole animals were harvested for our analysis, it is conceivable that the modest ∼2 fold increase in *RNase X25* mRNA levels we observed reflects a tissue specific differential response to WGA. Higher levels of *RNase X25* mRNAs may accumulate in tissues of the gut, with stable expression levels in remaining tissues. This effect could also explain the discrepancies in results observed in several high throughput analyses of starved *Drosophila* larvae or adults (see Table S1). This “dilution effect” may also explain the difference between the *Atg5* levels of expression observed in our experiment and the experiments of Barth et al. [46], who isolated ovaries for their analyses.

In addition to the housekeeping role, RNase T2 enzymes have acquired novel functions during eukaryote evolution. In some cases, novel functions appeared after gene duplications. This seems to be the case for plant RNase T2 enzymes that participate in defense mechanisms, and also for S-RNases, specialized T2 enzymes that determine gametophytic self-incompatibility in several plant species [12], [13]. In other cases, a single protein can have multiple roles. For example, both RNase activity-dependent and -independent functions have been proposed for human RNASET2. Lack of RNASET2 causes cystic leukoencephalopathy in humans and a similar phenotype in zebrafish [6], [47]. This neurological disorder is likely caused by lysosomal malfunction due to the high levels of rRNA that accumulate in these organelles when the enzyme is absent [6]. In addition, human RNASET2 has been shown to have anti-metastatic properties independent of its catalytic activity [48]. Another enzyme with more than one function is yeast Rny1. This protein may work in rRNA recycling during ribophagy-like processes, given its localization in vacuoles in normal growth conditions [9]. Additionally, Rny1 is responsible for tRNA cleavage during the cell's response to oxidative stress, after the enzyme is likely released from the vacuole into the cytoplasm. The accumulation of stable tRNA halves is thought to act as a signal during the stress response [9], [10]. Moreover, during the oxidative stress response, Rny1 is able to promote cell death through an unknown mechanism that is independent of its RNase activity [9]. Cleavage of tRNAs in stress conditions that inhibit cell growth is a response conserved in plants and animals [10], [49], and there is some evidence that tRNA fragments also accumulate in *Drosophila* [50], [51]. We showed here that *RNase X25* expression is increased in flies subjected to oxidative stress, making this enzyme the logical candidate for a role in tRNA cleavage in these insects, and suggesting that RNase X25 may have dual function, as shown for other members of the RNase T2 family.

Our phylogenetic analyses showed that most insects have only one gene belonging to the RNase T2 family in their genomes. However, parasitoid wasps of the genus *Nasonia* seem to be the exception. In these insects, several gene duplications have occurred, and there is EST evidence indicating that the duplicated genes are expressed. In addition, mutations in the conserved active site of these proteins suggest that the duplicated enzymes have attenuated activity. Interestingly, parasitoid wasps from other families have a symbiotic relationship with polydnaviruses that provide protein factors essential for parasitism [40], including proteins belonging to the RNase T2 family [52]. These virus-encoded RNase T2 proteins are expressed in the parasitoid larva and likely delivered into the host during the parasitization process [52]. Expression of the viral RNase alone in the host larva resulted in reduction in hemocyte populations and increase in susceptibility of the larva to bacterial and baculovirus infections, indicating that these RNases have an important immunosuppressive function during parasitism [41], [53]. *Nasonia* wasps do not have a polydnavirus symbiont. It is possible, then, that upon duplication of the conserved housekeeping *RNase T2* gene, the new proteins diverged and were recruited for a novel function in immunosuppression during parasitism, in a case of convergent evolution.

It is intriguing that the duplicated *Nasonia* RNases have mutations in the enzymes' active site. These changes have been previously reported in other proteins of the RNase T2 family, and it has been speculated that they may result in attenuated yet active enzymes [13], [14]. However, the biological significance of this mutation is not yet understood. Other RNase T2 proteins are also able to modulate immune responses. E^rns^, a Bovine Viral Diarrhea Virus RNase T2 protein, can inhibit the host beta interferon response potentially by interfering with the dsRNA signal [54], or through an intracellular mechanism involving cell-to-cell signaling even in the absence of virion particles [55]. This inhibitory effect on the beta interferon response depends on the RNase activity of E^rns^ [54], [55]. However, a catalysis-independent cytotoxic effect for this protein has also been proposed based on the ability of mutant proteins without RNase activity to induce cell death in swine kidney cells [56]. Omega-1, a secreted RNase T2 protein from *Schistosoma mansoni*, also has a cytotoxic effect on its host hepatic cells [57]. This protein is also able to induce a strong Th2-polarized immune response in the host, which is necessary for the efficient passage of parasite eggs from the intravascular sites of deposition to the intestinal or bladder lumen [58]. In this case, modulation of the immune response seems to be caused by suppression of protein synthesis after internalization by dendritic cells following recognition of the glycosylation signature of omega-1 [59]. The RNase activity of omega-1 is necessary for this immunomodulating role. However, other *Schistosoma* species appear to have inactive RNase T2 proteins in addition to enzymes with conserved active sites. It is possible that these proteins could also play a role in immunoregulation, although these inactive proteins are present also in free living planarians.

Recent insights from *Arabidopsis thaliana*, zebrafish, and human indicate that the RNase T2 enzymes carry out an important housekeeping function in normal cells [6], [7]. Arabidopsis mutants lacking this conserved RNase T2 activity accumulate RNA, mainly in the vacuole, have an increased rRNA half-life, and exhibit constitutive autophagy [7]; while *rnaset2* mutant zebrafish show aberrant accumulation of undigested rRNA in neuronal lysosomes and present brain lesions similar to those observed in leukocephalopathies associated with deficiencies in RNASET2 in humans [6], [47]. Thus, RNase T2 enzymes participate in the normal recycling of rRNA, and this housekeeping function seems to be essential for cellular homeostasis. Duplication and divergence of the RNase T2 gene family has occurred in the evolution of plants and fishes. Secreted RNases of the RNase A family seem to have acquired in vertebrates, including humans, some of the biological roles carried out by RNase T2 enzymes in other systems (reviewed by MacIntosh [2]). Use of the *Drosophila* model, whose genome encodes only one *RNase T2* gene and lacks RNase A homologs or other evident secretory RNases with similar activity, is likely to provide insight into the ancestral physiological function of this gene family in multicellular animals during normal growth and development, and also under stress conditions. Analyses of *Drosophila* mutants with reduced RNase X25 activity may lead to identification of phenotypic characteristics that could be the basis of genetic modifier screens to identify other key genes that participate in RNase T2 function. These may also prove important to understand how RNA degradation systems interface with other cellular processes.

## Supporting Information

Figure S1 **Expression profile of** ***RNase X25*** **in different adult tissues from the modENCODE database.**
(PDF)

Figure S2 **Expression profile of** ***RNase X25*** **in different adult and larval tissues from the FlyAtlas database.**
(PDF)

Table S1 **Regulation of RNase X25 expression under nutritional stress or other conditions, obtained from public databases or the literature.**
(PDF)

Table S2 **Proteins belonging to the RNase T2 family used for phylogenetic analyses.**
(XLSX)
