# Supplementary material for: Phylogenetic Analyses and Characterization of RNase X25 from Drosophila melanogaster Suggest a Conserved Housekeeping Role and Additional Functions for RNase T2 Enzymes in Protostomes
Source: PLoS One. 2014 Aug 18;9(8):e105444. doi: 10.1371/journal.pone.0105444 (PMC4136927; doi:10.1371/journal.pone.0105444)
Supplement: Figure S1 — Expression profile of RNase X25 in different adult tissues from the modENCODE database. (PDF) [file pone.0105444.s001.pdf]

Supplemental Figure S1

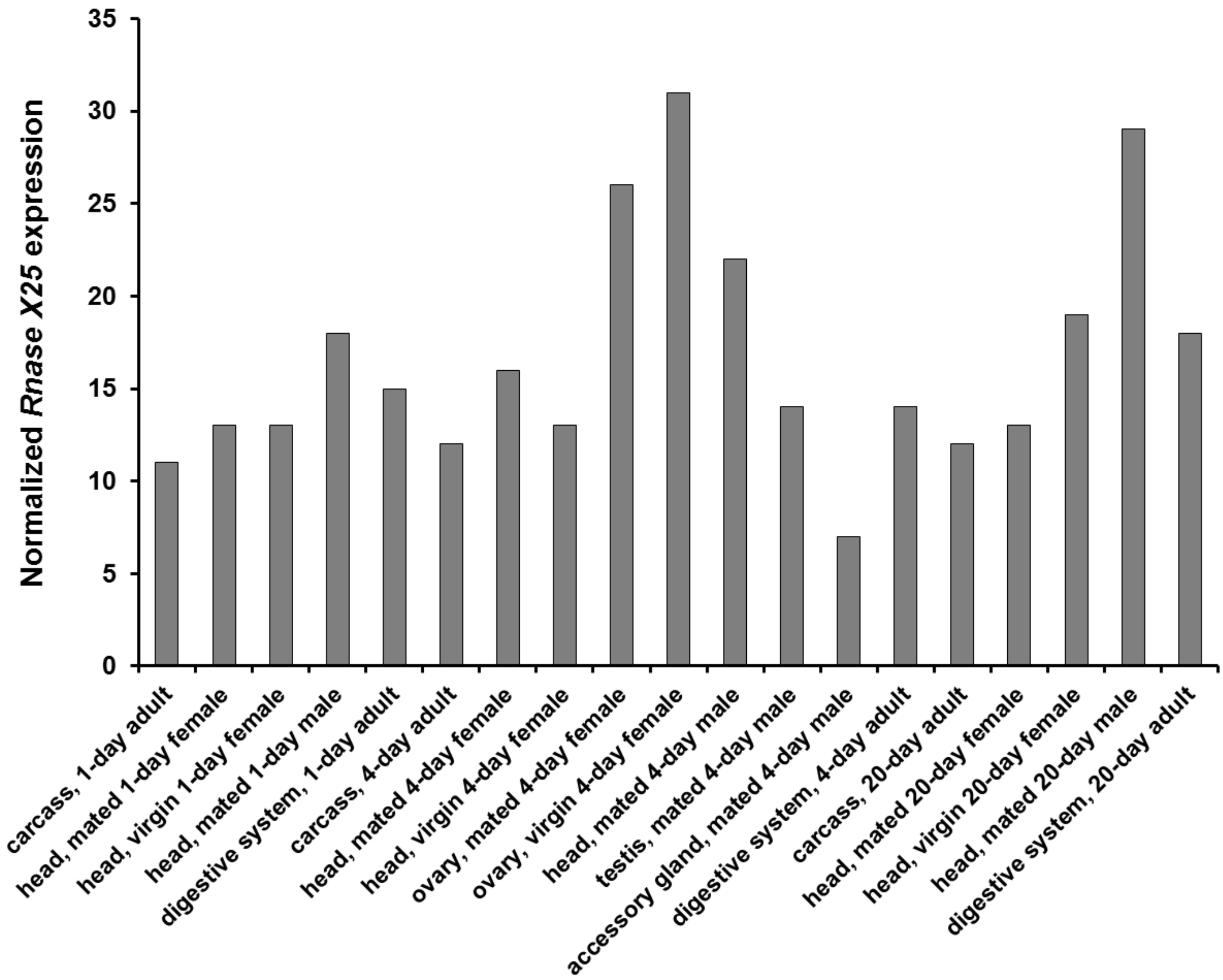

Supplemental Figure S1. Expression profile of *RNase X25* in different adult tissues, obtained from the modENCODE database through a query in FlyBase (http://flybase.org). Data were obtained through RNA-Seq analysis of the tissues described in the figure, and were reported as RPKM (Reads per kilo base per million).
