# Supplementary material for: Phylogenetic Analyses and Characterization of RNase X25 from Drosophila melanogaster Suggest a Conserved Housekeeping Role and Additional Functions for RNase T2 Enzymes in Protostomes
Source: PLoS One. 2014 Aug 18;9(8):e105444. doi: 10.1371/journal.pone.0105444 (PMC4136927; doi:10.1371/journal.pone.0105444)
Supplement: Figure S2 — Expression profile of RNase X25 in different adult and larval tissues from the FlyAtlas database. (PDF) [file pone.0105444.s002.pdf]

Supplemental Figure S2

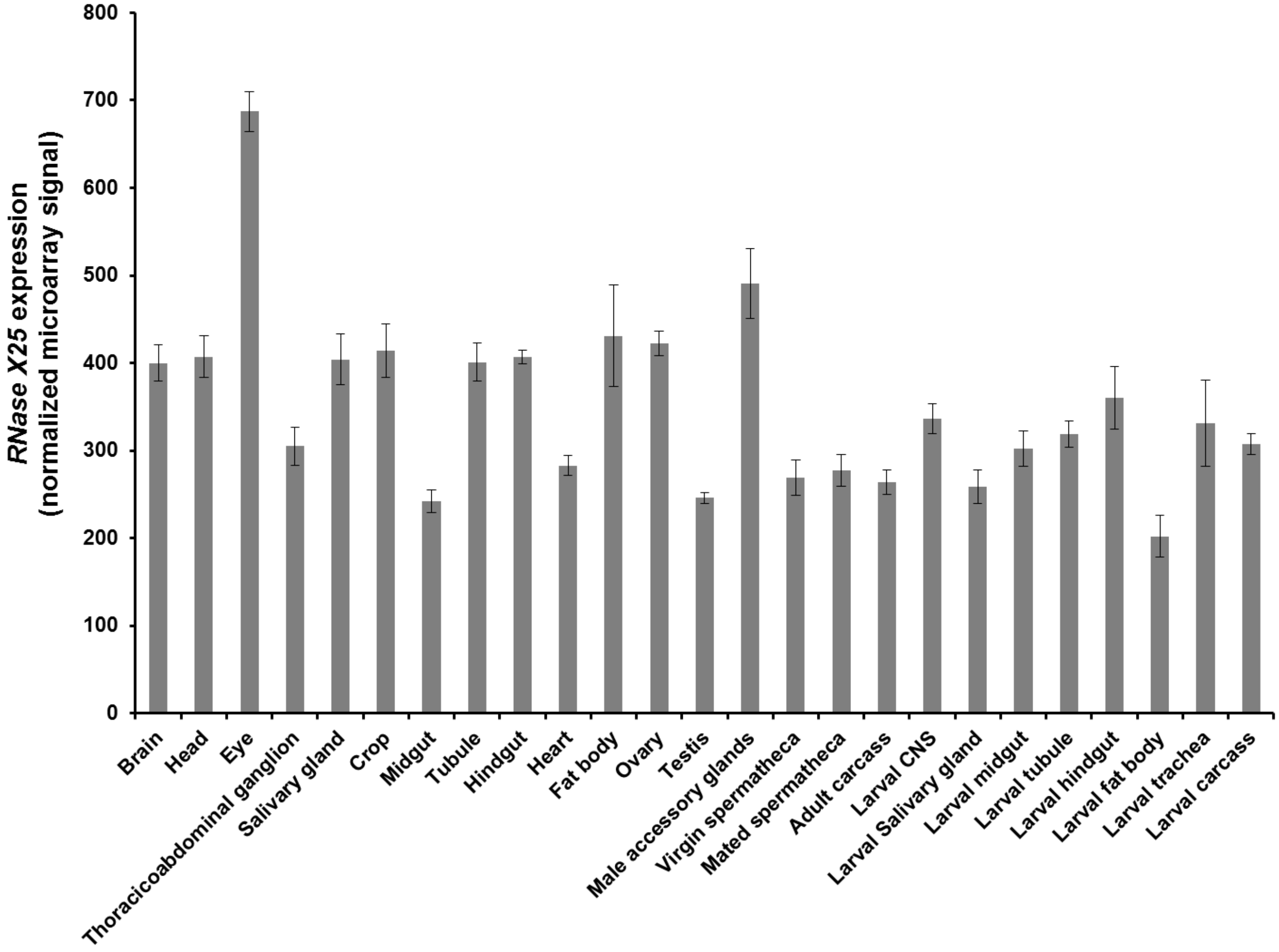

Supplemental Figure S2. Expression profile of *RNase X25* in different adult and larval tissues, obtained from the FlyAtlas database (http://flyatlas.org). Data were obtained through Affymetrix microarray chip analysis of the tissues described in the figure, and were reported as average normalized signal intensity (4 chips per sample) +/- standard error.
