# Supplementary material for: Phylogenetic Analyses and Characterization of RNase X25 from Drosophila melanogaster Suggest a Conserved Housekeeping Role and Additional Functions for RNase T2 Enzymes in Protostomes
Source: PLoS One. 2014 Aug 18;9(8):e105444. doi: 10.1371/journal.pone.0105444 (PMC4136927; doi:10.1371/journal.pone.0105444)
Supplement: Table S1 — Regulation of RNase X25 expression under nutritional stress or other conditions, obtained from public databases or the literature. (PDF) [file pone.0105444.s003.pdf]

**Supplemental Table S1**. Regulation of RNase X25 expression under nutritional stress or other conditions, obtained from public databases or the literature

| Sample source | Treatment/condition | Expression | Reference |
|---|---|---|---|
| 2nd instar larvae | Fed w/ sugar | No Change[1] | Zinke et al., 2002 |
| 2nd instar larvae | Starvation | No Change[1] | Zinke et al., 2002 |
| *y w* flies | Fed w/ yeast | Downregulated[2] | Gershman et al., 2007 |
| 3rd instar *W^1118* larvae | Fed w/ wheat germ agglutinin | Upregulated | Li et al., 2009 |
| 3rd instar *W^1118* larvae | Starvation | No Change | Li et al., 2009 |
| 2nd instar larvae | Starvation | Upregulated | Palanker et al., 2009 |
| Oregon R adults | Fed w/ pyrethrum | Upregulated | Jensen et al., 2006 |
| Cultured Kc167 cells | Treated w/ Ecdysone | Downregulated | Akdemir et al., 2007 |
| Cultured Kc167 cells | Exposed to γ-ray | Upregulated | Akdemir et al., 2007 |

**Footnotes**

[1] Only transcripts with 4 fold or more change in expression were reported.

[2] In this experiment, flies were yeast-deprived for 4 days and then refed with yeast-containing media. Thus, downregulation upon refeeding could indicate upregulation of *RNase X25* during the low nutrient period.

**References**


Akdemir F, Christich A, Sogame N, Chapo J, Abrams JM (2007) p53 directs focused genomic responses in Drosophila. *Oncogene* 26: 5184-5193.

Gershman B, Puig O, Hang L, Peitzsch RM, Tatar M, Garofalo RS (2007) High-resolution dynamics of the transcriptional response to nutrition in *Drosophila*: a key role for dFOXO. *Physiol Genomics* 29: 24-34.

Jensen HR, Scott IM, Sims SR, Trudeau VL, Arnason JT (2006) The effect of a synergistic concentration of a *Piper nigrum* extract used in conjunction with pyrethrum upon gene expression in *Drosophila melanogaster*. *Insect Mol Biol* 15: 329-339.

Li HM, Sun L, Mittapalli O, Muir WM, Xie J, Schemerhorn BJ, Sun W, Pittendrigh BR, Murdock LL (2009) Transcriptional signatures in response to wheat germ agglutinin and starvation in *Drosophila melanogaster* larval midgut. *Insect Mol Biol* 18: 21-31.

Palanker L, Tennessen JM, Lam G, Thummel CS (2009) *Drosophila* HNF4 regulates lipid mobilization and beta-oxidation. *Cell Metab* 9: 228-239.

Zinke I, Schutz CS, Katzenberger JD, Bauer M, Pankratz MJ (2002) Nutrient control of gene expression in *Drosophila*: microarray analysis of starvation and sugar-dependent response. *EMBO J* 21: 6162-6173
